# Long-term forecasting of a motor outcome following rehabilitation in chronic stroke via a hierarchical bayesian dynamic model

**DOI:** 10.1186/s12984-023-01202-y

**Published:** 2023-06-29

**Authors:** Nicolas Schweighofer, Dongze Ye, Haipeng Luo, David Z. D’Argenio, Carolee Winstein

**Affiliations:** 1 Biokinesiology and Physical Therapy, University of Southern California, Los Angeles, USA; 2 Computer Science, University of Southern California, Los Angeles, USA; 3 Biomedical Engineering, University of Southern California, Los Angeles, USA

**Keywords:** Chronic stroke, Neurorehabilitation, Motor learning, Forecasting model, Hierarchical bayesian modeling, Dynamical models

## Abstract

**Background:**

Given the heterogeneity of stroke, it is important to determine the best course of motor therapy for each patient, i.e., to personalize rehabilitation based on predictions of long-term outcomes. Here, we propose a hierarchical Bayesian dynamic (i.e., state-space) model (HBDM) to forecast long-term changes in a motor outcome due to rehabilitation in the chronic phase post-stroke.

**Methods:**

The model incorporates the effects of clinician-supervised training, self-training, and forgetting. In addition, to improve forecasting early in rehabilitation, when data are sparse or unavailable, we use the Bayesian hierarchical modeling technique to incorporate prior information from similar patients. We use HBDM to re-analyze the Motor Activity Log (MAL) data of participants with chronic stroke included in two clinical trials: (1) the DOSE trial, in which participants were assigned to a 0, 15, 30, or 60-h dose condition (data of 40 participants analyzed), and (2) the EXCITE trial, in which participants were assigned a 60-h dose, in either an immediate or a delayed condition (95 participants analyzed).

**Results:**

For both datasets, HBDM accounts well for individual dynamics in the MAL during and outside of training: mean RMSE = 0.28 for all 40 DOSE participants (participant-level RMSE 0.26 ± 0.19—95% CI) and mean RMSE = 0.325 for all 95 EXCITE participants (participant-level RMSE 0.32 ± 0.31), which are small compared to the 0-5 range of the MAL. Bayesian leave-one-out cross-validation shows that the model has better predictive accuracy than static regression models and simpler dynamic models that do not account for the effect of supervised training, self-training, or forgetting. We then showcase model’s ability to forecast the MAL of “new” participants up to 8 months ahead. The mean RMSE at 6 months post-training was 1.36 using only the baseline MAL and then decreased to 0.91, 0.79, and 0.69 (respectively) with the MAL following the 1st, 2nd, and 3rd bouts of training. In addition, hierarchical modeling improves prediction for a patient early in training. Finally, we verify that this model, despite its simplicity, can reproduce previous findings of the DOSE trial on the efficiency, efficacy, and retention of motor therapy.

**Conclusions:**

In future work, such forecasting models can be used to simulate different stages of recovery, dosages, and training schedules to optimize rehabilitation for each person.

*Trial registration* This study contains a re-analysis of data from the DOSE clinical trial ID NCT01749358 and the EXCITE clinical trial ID NCT00057018

**Supplementary Information:**

The online version contains supplementary material available at 10.1186/s12984-023-01202-y.

## Introduction

Recent modeling work has sought to predict the long-term spontaneous recovery of individuals post-stroke from baseline clinical or neural data, e.g., [1–4]. Whereas such predictions are useful for clinical and research stratification, the neurorehabilitation clinician needs to accurately predict the long-term changes in motor outcomes in response to specific treatments. With the predicted responses, the clinician could then determine the best course of motor therapy for each patient, i.e., personalize rehabilitation [5].

A difficulty is that stroke is heterogeneous and exhibits considerable variability, including in response to motor therapy [6]. It is known that the integrity of the corticospinal tract predicts gains in functional outcomes due to rehabilitation, e.g., [4, 7, 8]. However, multiple other factors are also likely to affect these gains, as well as the retention of these gains following rehabilitation. For instance, we have previously shown that the integrity of visuospatial working memory modulated the effect of blocked, but not distributed, training schedules in chronic stroke [9]. In addition, in re-analyses of the data of the EXCITE [10] and DOSE [11] trials, we have shown that approximately one-fourth of participants continued to see improvements in upper extremity (UE) function following training; conversely, another fourth lost most gains in UE function that resulted from therapy [12, 13].

Given this variability in response to therapy, we need a paradigm shift in predictive modeling in neurorehabilitation that, in addition to clinical and lesion data, incorporates repeated measurements of motor outcomes as soon as they become available during motor therapy. Predictive models that primarily consider such repeated measurements indexed in time order (i.e., time-series) are *forecasting models*. For example, a recent model can more accurately forecast spontaneous recovery 6 months post-stroke when incorporating repeated measurements than only baseline data [14]. Here, we extend such an approach to forecast the effect of rehabilitation in chronic stroke.

What should be the form of forecasting models in neurorehabilitation? Since neurorehabilitation is based on the premise that sensorimotor activity improves motor recovery via brain plasticity, i.e., “changeability”, the models need to account for the changes in outcomes both during movement therapy, when an increase in performance is expected, and outside of therapy, when both a decrease in performance due to forgetting and an increase in performance are possible. Previously, we proposed a piece-wise linear model of changes in a motor outcome in the DOSE clinical trial, in which the periods of therapy marked the limit between the different linear segments [13]. Although this model well accounted for positive and negative changes both during and following therapy, a model of this type cannot generalize to other datasets because it depends on the timing of training and measurements.

We propose a state-space modeling approach to predict motor outcomes during and following rehabilitation post-stroke. The model has a compact representation and an adjustable time resolution, allowing generalization to different data sets and even to different schedules of therapy for individual patients. The model extends a previous non-linear, first-order state-space model that explained the long-term changes, and the variability in these changes, in arm use following training in the EXCITE trial [12]. This previous model uses a retention term to account for the performance decay often observed post-training, at least in subgroups of patients [12, 13] and a “self-training” term to account for the change in spontaneous use of the paretic limb outside of training when UE function is above a threshold post-therapy [12, 13, 15, 16], which further increases future use and function. In the present model, we further account for the response to therapy via an input term proportional to the dose of motor training, as in our previous piece-wise model [13]. Indeed, animal studies, meta-analyses, and recent clinical trials with large doses, including the DOSE trial, showed that large training doses improve UE function, e.g., [11, 17–19].

Previous models in neurorehabilitation typically predict the mean of the future outcome, e.g., [8, 20, 21]. However, such point estimation of a future outcome is insufficient for clinical decision-making in neurorehabilitation because clinicians need to account for the uncertainty of the forecast when assessing different treatment options.^1^ To provide interval estimation, we utilize the Bayesian approach, which extends our previous work [12], as Bayesian models naturally deal with uncertainties by focusing on the probability distributions of *all* parameters.

A final difficulty for accurate long-term forecasting in neurorehabilitation, however, is that for each new patient, there is initially no or little data on the effect of motor therapy. A hierarchical Bayesian model [22] can, in theory, refine the initial predictions by incorporating prior information from similar patients, via “hyper-parameters.” Crucially, these hyper-parameters can be used as individual prior parameters when predicting the response of a new individual when little outcome data are available, i.e., early in therapy.

Here, we therefore propose and test a novel hierarchical Bayesian dynamic modeling (HBDM) framework that can accurately forecast a clinical measure following rehabilitation in chronic stroke. As a testbed of our model, we use the Motor Activity Log (MAL) data from both the DOSE trial [11] and the EXCITE trial [10]. We test whether a minimal model with three terms, accounting for retention, response to external training, and self-learning, respectively, can better predict the MAL than reduced dynamical models and non-dynamical regression models for these two datasets. Then, using the DOSE data, we simulate the model to forecast the MAL of “new” patients up to 8 months ahead and study the change in the long-term accuracy of the forecasts as additional training data becomes available. We compare the prediction accuracy for models with and without a hierarchical structure for different ranges of forecasting. Finally, we validate the model by testing whether it can account for our previous results on the DOSE dataset on the efficacy, efficiency, and retention of motor training in chronic stroke.

## Methods

### Participants and data

We first developed and validated the model with the MAL data of 40 participants enrolled in the DOSE trial conducted at the University of Southern California [11]. The participants had mild-to-moderate upper extremity motor impairment chronically after stroke (onset at least 5-month before inclusion). Participants were randomly assigned to the 0, 15, 30, and 60-h doses, with the dosages distributed over 3 week-long training bouts, separated by 1 month. The intervention was based on the Accelerated Skill Acquisition Program (ASAP) [23], which includes elements of skill acquisition through challenging and progressive task practice. The MAL Quality of Movement (QOM) subscale, which measures the participant’s perception of the amount and quality of functional motor tasks by asking them to recall and rate the quality of movement of the paretic arm for 28 activities of daily living [24], was collected by blinded assessors in 14 longitudinal assessments administered: (1) twice before the first training bout; (2) immediately before and after each of the three 1-week training bouts; and (3) monthly for 6 months following the last training bout.

We further validated the model with the data from the EXCITE trial conducted at 7 US sites, in which participants who had a first stroke within the previous 3 to 9 months and with mild-to-moderate impairment were randomly assigned to either an immediate or a delayed Constraint Induced Movement Therapy (CIMT) group [10, 25]. The immediate group received 10 days of therapy from inclusion; the delayed group received 10 days of therapy after a one-year delay. Participants were tested with the MAL QOM, recorded by blinded assessors, immediately before and after the scheduled therapy for both groups, and at 4 months, 8 months, 16 months, 20 months, and 24 months, with a maximum of 9 data points were available. Due to the limited MAL data per person, we excluded participants with missing observations, resulting in 50 and 45 participants in the immediate and delayed groups, respectively.

### The hierarchical bayesian dynamic model (HBDM)

We developed a hierarchical Bayesian dynamic model (HBDM) of the changes in the MAL both during training and outside of training post-stroke. The model has three levels: an individual measurement level (level 1), a subject level (level 2), and a population level (level 3).

#### Level 1: Modeling intra-individual variations (over time)

In level 1, we modeled how the MAL is updated at each time *t* for each subject *i*. The model contains a single state-space equation of motor memory, a sigmoidal function that maps a motor memory to a predicted MAL, and an observation model that accounts for the measurement noise in the MAL. The motor memory (i.e., the state of the system) ${x}_{i}^{t}$ and the predicted MAL ${m}_{i}^{t}$ are updated by the following equations:

1 $${x}_{i}^{t+1}=\left\{\begin{array}{l}{\alpha }_{i}{x}_{i}^{t}+{{\beta }_{i}u}_{i}^{t}\quad{ \text{if}\, \text{u}}_{i}^{t} >0, \\ {{\upalpha }}_{i}{x}_{i}^{t}+{{\upgamma }}_{i}{m}_{i}^{t} \quad \text{else}. \end{array}\right.$$

2 $${m}_{i}^{t}=10/\left(1+{e}^{-0.2{x}_{i}^{t} }\right)-5$$

where $$0 \le {\alpha }_{i}\le 1$$ is a retention rate parameter (such that $${\alpha }_{i}$$ close to 1 corresponds to little forgetting), $${\beta }_{i}\ge 0$$ a learning rate parameter, and $${\gamma }_{i}$$$$\ge 0$$ controls the strength of self-training. By passing the motor memory through the sigmoidal function (Eq. 2), the predicted MAL $${m}_{i}^{t}$$ always lies in the proper 0–5 range of the MAL. In the DOSE trial, $${u}_{i}^{t}=$$ [0, 5, 10, 20] + 2 is one of the four doses for each of the three weeks of training, including two additional hours of movement testing [13] (as a reminder, the total nominal doses are 0, 15, 30, and 60 h). In EXCITE, $${u}_{i}^{t}=$$ 60, given upon inclusion (immediate group) or 1 year later (delayed group). The constant sigmoid slope of $$0.2$$ was found in preliminary model fitting. Note that no process (state) noise was included, because the data are scarce [26].

To account for outliers, the observed MAL (${\text{M}\text{A}\text{L}}_{i}^{t}$) is modeled with a generalized Student’s *t*-distribution; that is:

3 $${\text{M}\text{A}\text{L}}_{i}^{t} \sim \text{S}\text{t}\text{u}\text{d}\text{e}\text{n}\text{t}\text{T}\left({m}_{i}^{t}, { \sigma }_{\text{M}\text{A}\text{L}}, \nu \right).$$

where ${m}_{i}^{t}$ (the predicted MAL) is the center of the distribution, ${\sigma }_{\text{M}\text{A}\text{L}}$ is a scale parameter, and $\nu$ is the degree of freedom. Note that ${\sigma }_{\text{M}\text{A}\text{L}}$ is subject-independent because we assume the measurement noise is an inherent property of the MAL.

#### Level 2: Modeling inter-individual variations

In level 2, to account for the differences between individuals, the model parameters are assumed to be random variables following different probability distributions for each participant. Thus, in level 2, we model the individual parameters ${{\alpha }}_{i}$, ${{\beta }}_{i }, {\gamma }_{i}$, as well as the initial state ${x}_{i}^{0}$ with the following prior distributions:

$${\alpha }_{i} \sim 1/\left(1+{e}^{-\mathcal{N}\left({\theta }_{\alpha }, { \sigma }_{\beta } \right)}\right)$$

$${\beta }_{i} \sim {\mathcal{N}}_{\left[0,{\infty }\right)}\left({\theta }_{\beta }, {\sigma }_{\beta }\right)$$

$${\gamma }_{i} \sim {\mathcal{N}}_{\left[0,{\infty }\right)}\left({\theta }_{\gamma },{\sigma }_{\gamma }\right)$$

where $\mathcal{N}\left(\mu ,\sigma \right)$ is the normal distribution with mean $\mu$ and standard deviation $\sigma$, the retention rate ${{\alpha }}_{i}$ is constrained between 0 and 1 via the sigmoid function, and the learning rates ${\beta }_{i }$ and self-training rates ${\gamma }_{i}$ are non-negative with truncated normal distributions $\left({\mathcal{N}}_{\left[0,{\infty }\right)}\right)$. As in our previous model [13], the baseline $\text{M}\text{A}{\text{L}}_{\text{i}\text{n}\text{i}}$is a (linear) covariate of the initial memory state:

$${x}_{i}^{0}\sim{\mathcal{N}}_{\left[0,{\infty }\right)}\left(k \text{M}\text{A}{\text{L}}_{\text{i}\text{n}\text{i}},{\sigma }_{\text{i}\text{n}\text{i}}\right)$$

#### Level 3: Modeling the population level

Finally, in the level 3 of the hierarchy, the hyper-parameters, ${\theta }_{{\upalpha }}$, ${\theta }_{{\upbeta }}$, ${\theta }_{\gamma }$, ${{\sigma }}_{{\alpha }}$, ${{\sigma }}_{{\beta }}$, ${\sigma }_{\gamma }$, ${\sigma }_{\text{i}\text{n}\text{i}}$, ${\sigma }_{\text{M}\text{A}\text{L}}$, $k$, and $\nu$ govern the prior distributions of the individual parameters. The hyper-parameters are initially sampled from weakly-informative prior distributions as shown in Table 1. We use normal priors for the location hyper-parameters ${\theta }_{}$ for better explainability and to impose weak regularizations on relevant parameters, a truncated normal prior on the slope coefficient *k* to ensure positivity, and inverse-gamma priors for the scale hyper-parameters ${\sigma }_{}$, which tend to drive parameters further away from zero than truncated-normal priors.

### Bayesian parameter estimation methods

Parameter estimation for HBDM involves simultaneous determination of the population parameters, as well as the individual-level parameters given all the data from all participants. Fitting the dynamic model given by Eqs. 1–3 was performed via Bayesian inference, which incorporates the prior distributions and data to generate a posterior distribution for each random variable via the Bayes’ rule. For each dataset, the HBDM fits the data from all participants simultaneously using the software Stan (via the RStan interface) [27].

We imposed weakly-informative priors on the hyper-priors. In particular, the priors on ${\mu }_{\alpha }$ and ${\sigma }_{\alpha }$, to reflect the prior knowledge that the median retention rate is about 0.86 as found in our previous work with the DOSE dataset [13]. Similarly, the hyper-priors for the learning rate and self-training rate are selected based on the ranges of the parameters found in our previous studies [12] with the EXCITE dataset and [13] with the DOSE dataset—see Table 1.

Note that the MAL measurements are non-evenly spaced. In DOSE, the smallest spacing is 1 week and in EXCITE, the smallest spacing is 2 weeks. To compare the hyper-parameters in both datasets, we used a time-step of 1 week for both datasets and considered non-available data as missing. The Bayesian method smoothly deals with the issue of missing data.

**Table 1:** Subject-dependent parameters and population hyper-priors used in the best model

Model parameters	Priors	Hyper-priors
Retention rate	${\alpha }_{i} \sim 1/\left(1+{e}^{-\mathcal{N}\left({\theta }_{\alpha }, { \sigma }_{\alpha } \right)}\right)$	${\theta }_{\alpha } \sim \mathcal{N}\left(\text{2,1}\right)$ ${\sigma }_{\alpha } \sim \text{I}\text{n}\text{v}\text{G}\text{a}\text{m}\text{m}\text{a}\left(3, 2\right)$
Learning rate	${\beta }_{i } \sim {\mathcal{N}}_{\left[0,{\infty }\right)}\left({\theta }_{\beta }, {\sigma }_{\beta }\right)$	${\theta }_{\beta } \sim \mathcal{N}\left(0, 1\right)$ ${\sigma }_{\beta } \sim \text{I}\text{n}\text{v}\text{G}\text{a}\text{m}\text{m}\text{a}\left(4, 2\right)$
Self-training rate	${\gamma }_{i} \sim {\mathcal{N}}_{\left[0,{\infty }\right)}\left({\theta }_{\gamma },{\sigma }_{\gamma }\right)$	${\theta }_{{\upgamma }} \sim \mathcal{N}\left(0, 1\right)$ ${{\sigma }}_{{\gamma }} \sim \text{I}\text{n}\text{v}\text{G}\text{a}\text{m}\text{m}\text{a}\left(4, 2\right)$
Initial state of memory	${x}_{i}^{0} \sim{ \mathcal{N}}_{\left[0,{\infty }\right)}\left(k \text{M}\text{A}{\text{L}}_{\text{i}\text{n}\text{i}}, {\sigma }_{\text{i}\text{n}\text{i}}\right)$	$k \sim {\mathcal{N}}_{\left[0,{\infty }\right)}\left(0, 2\right)$ ${\sigma }_{\text{i}\text{n}\text{i}} \sim \text{I}\text{n}\text{v}\text{G}\text{a}\text{m}\text{m}\text{a}\left(3, 2\right)$

Data	Likelihood	Hyper-priors
MAL (measured)	$\text{S}\text{t}\text{u}\text{d}\text{e}\text{n}\text{t}\text{T}\left({\text{M}\text{A}\text{L}}_{i}^{t} | {m}_{i}^{t}, { \sigma }_{\text{M}\text{A}\text{L}}, \nu \right)$	$\nu \sim \text{G}\text{a}\text{m}\text{m}\text{a}\left(2, 0.1\right)$ ${\sigma }_{\text{M}\text{A}\text{L}} \sim {\mathcal{N}}_{\left[0,{\infty }\right)}\left(0.25, 0.1\right)$

Note that we modeled the measured $\text{MAL}_{i}^{t}$ with a generalized $t$-distribution centered at ${m}_{i}^{t}$ with a scale parameter ${ \sigma }_\text{MAL}$ and the degrees of freedom $\nu$

We ran the No-U-Turn sampler implemented in Stan with 6 chains for 10,000 iterations, including 5000 warm-up samples. We verified convergence with the following methods. First, we checked that the *improved R-hat* was below the recommended threshold of 1.01 [28] for all parameters in the models for both the DOSE and the EXCITE datasets. The low R-hats suggest that the parallel simulation chains are well-mixed and have converged to the target posterior distribution. Second, we checked the prior posterior overlap (PPO). Almost all parameters in our models (for both datasets) have PPO below the 35% threshold, which indicates adequate parameter identifiability [29]. The only exceptions are individual learning rates for subjects D5, D7, D8, and D9 with PPOs between 35% and 40% (an acceptable range).

Finally, RStan automatically detects and warns users of various potential issues with the algorithm [30], notably regarding the estimated effective sample size (ESS). Upon termination, our models received no runtime warnings from RStan.

We then used the *MCMCvis* package and functions from RStan to generate summary statistics and plots of the posterior distributions for the parameters in our model. A “leave-one-subject-out” cross-validation experiment (described below) was performed to evaluate the model’s ability to forecast individual future outcomes using the high-performance-computing cluster. To validate the model structure, we compared the full HBDM with simpler models, dropping each of the retention, learning, and self-training terms in Eq. 1. We then compared the full model to a model without random effects (no between-subject variability). Finally, we compared the full dynamic model to static linear and logistic regression models, in which the state-space Eq. (1) is replaced by a simple linear function of time (i.e., weeks):

4 $${x}_{i}^{t}={c}_{i}+{d}_{i} t$$

where ${c}_{i},{d}_{i}$ are estimated coefficients (for subject *i)* and *t* is the number of weeks after the initial MAL measurement. Additionally, the logistic model contains the sigmoidal function (Eq. 2) that converts motor memory into predicted MAL.

Model comparison (between the best model and simpler models) was performed using WAIC (Watanabe-Akaike information criterion) and PSIS-LOO (Pareto smoothed importance sampling leave-one-out cross-validation) [31]. The WAIC is an estimation of expected log point-wise predictive density (ELPD) that adjusts for overfitting using the effective number of parameters. The ELPD is a theoretical(or ideal) measure of a model’s predictive accuracy on unseen data [31]. Maximizing ELPD is equivalent to minimizing the KL-divergence of the true data-generating process to the posterior predictive distribution.

PSIS-LOO provides a different estimate of ELPD, named *elpd_loo*, and allows us to compute the *elpd_diff*, a measure of the pairwise difference in *elpd_loo*. The *elpd_loo* has shown to be asymptotically equal to WAIC but also “more robust in the finite case with weak priors or influential observations” [31]. Note that when comparing several models, *elpd_diff* is only computed for each model against the best model (which has the largest *elpd_loo*). Then, under the normality assumption, we estimate the 95% confidence interval of *elpd_diff* and $Pr\left(better\right)$, the probability that a model is better than the best model found through PSIS-LOO (i.e., when *elpd_diff*$\ge 0$).

### Evaluation of forecasting accuracy on unseen data with the DOSE dataset

We then evaluated the long-term forecasting accuracy in four different scenarios on the DOSE dataset (see Results). We used a “leave-one-subject-out” simulated experiment, which repeatedly re-fits the model while masking a number of last MAL measurements for a “left-out” participant. This procedure allows us to examine the model’s ability to utilize a database of “past” participants to predict the outcome of a “new” participant (with few data). We also compared the full model with a model without the hierarchical structure. We quantified the model’s forecasting accuracy using a modified RMSE, named Bayesian forecasting RMSE (BF-RMSE). The RMSE is the root of the average squared difference between the predicted and the observed MAL. When computing the RMSE using point estimates, we used the posterior medians of ${m}_{i}^{t}$ in Eq. (2) as the model’s predictions for patient *i*’s MAL at week *t*. The BF-RMSE, in contrast, evaulates an interval estimate against a point observation. In particular, the BF-RMSE is the RMSE between each posterior draw of the forecasted values and the corresponding masked measurements for each participant. We computed the mean BF-RMSE at each “future” time-point, by averaging over participants, and used the permutation test to estimate one-sided *p*-values, i.e., the probability that the non-hierarchical model has a lower mean BF-RMSE than the full model.

## Results

### Fitting the hierarchical bayesian state-space models

#### DOSE dataset

**Fig. 1:**
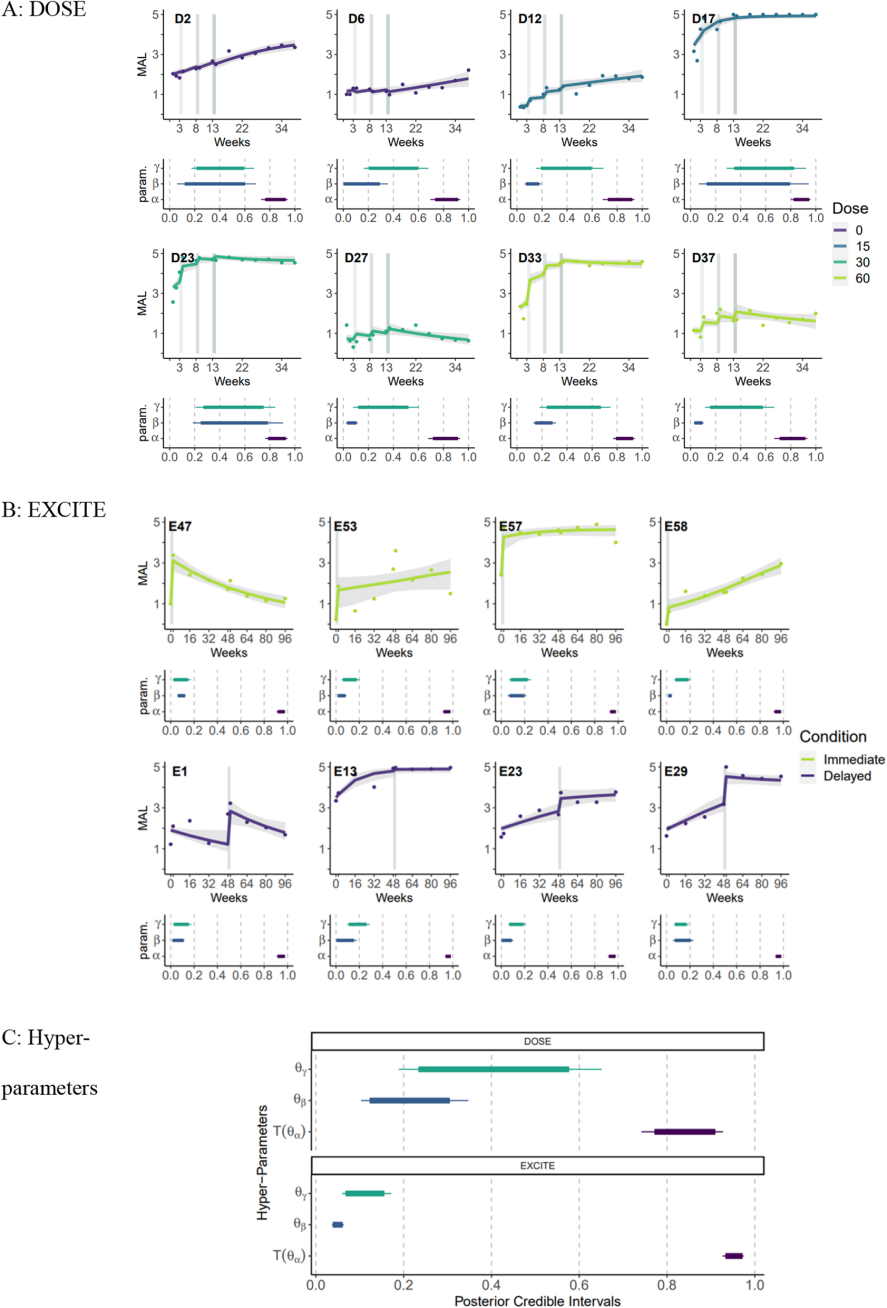
Data, model fit, and parameter estimates for the best learning model. **A **DOSE data: Example of data, model fit, and parameter estimates for eight participants arranged by doses of training. **B **EXCITE data: Example for eight participants arranged by the timing of training (immediate vs. delayed). Upper panel: MAL data and fit for the best model for each participant. The fit was overall excellent, with RMSE = 0.28 for all 40 participants (individual RMSE 0.26 $\pm$0.19). These examples also illustrate the large variability between participants. Variability in response to training is due to a low (e.g., D27) or high learning rate (e.g., D23). A combination of small learning and self-learning rates is highly detrimental for long-term performance (e.g., D27, D37), but the opposite yields improved long-term outcomes (e.g., D17). Dot: data. Lines: mean model fit. Shaded area: 95% CI. Lower panel: Posterior parameter distributions of the three main parameters (self-training rate ${\gamma }_{i}$, learning rate ${\beta }_{i }$, and retention rate ${\alpha }_{i})$. The thick bars show the 95% parameter CI and the thin bars the 99% CI. **C **Hyper-parameters: Posterior distributions for the corresponding hyper-parameters. Note that $\text{T}\left({{\theta }}_{{\alpha }}\right)$ is the transformed ${{\theta }}_{{\alpha }}$ parameter (via the sigmoid function), which corresponds to the median of the logit-normal prior distribution for individual retention rates (${{\alpha }}_{\text{i}}$)

Figure 1A shows examples of fits to the DOSE data for the best HBDM (Eqs. 1–3 in Methods) with the corresponding individual parameters (retention rates, learning rates, and self-training rates) for eight representative participants (two per dose). The model achieved good convergence, with R-hat < 1.01 for all parameters. The fit was overall excellent, with RMSE = 0.28 for all 40 participants (participant-level RMSE 0.26 $\pm$0.19—95% CI), which is less than 6% of the MAL 0–5 scale. The median retention rates for each subject were between 0.83 and 0.89, in line with the mean estimated retention rate in our previous work with the EXCITE dataset of 0.86 [12]. However, there was a large between-subject variability. Whereas several participants showed a weak training effect with a median learning rate of less than 0.1 (e.g., D27), some showed learning rates above 0.4 (e.g., D23). Whereas a combination of small learning and self-learning rates is highly detrimental for long-term performance (e.g., D27, D37), the opposite yields improved long-term outcomes (e.g., D17).

### EXCITE dataset

Figure 1B shows examples of fits of the same “best” model to the EXCITE data and parameters for eight participants (four for each group: immediate and delayed). Here, again, the model achieved good convergence, with R-hat < 1.01 for all parameters. The mean RMSE was 0.35 (participant-level RMSE $0.32\pm 0.31$), which is 7% of the MAL 0–5 scale. Thus, despite the few data points available per participant in EXCITE (9 instead of 14 in DOSE), the fit is still very good. Here again, there was large variability in the parameters between participants, notably for the effect of training. For instance, participants E47 and E1 showed pronounced decay outside of training, as captured by relatively small retention and self-training parameters. In contrast, participants whose MAL continues to increase post-training showed high self-training parameters. Finally, as for DOSE, training effects vary significantly between subjects (e.g., E57 and E29).

Comparison of the hyper-parameters between the two datasets (Fig. 1C) shows that, as a group, the EXCITE participants have smaller learning rates (95% CI 0.040–0.06 for 0.12–0.30), smaller self-learning rates (95% CI 0.068–0.15 vs. for DOSE: 0.23–0.57), and larger retention rates (95% CI 0.93–0.97, corresponding to a time constant of decay 15.2–34.6 weeks, vs. for DOSE: 0.77–0.90, time constant 3.4–11.0 weeks). We comment on the possible reasons for these differences in the Discussion.

We then tested for possible pair-wise correlations between the three parameters of Eq. 1, i.e., the retention rate ${\alpha }_{i} ,$ learning rate ${\beta }_{i}$ and self-training rate ${{\gamma }}_{i}$. We computed the 95% CI of the Pearson’s correlation coefficient with bootstrapping for 5000 iterations. As shown in Additional file 1: Fig. S1, all parameters are positively correlated for both clinical trials. The (relatively small) correlation between the learning rates ${\beta }_{i}$ and the self-training rates ${{\gamma }}_{i}$ suggests that participants respond similarly to both types of training (supervised and self-administered). The correlation between retention rates ${\alpha }_{i}$ and the self-training rates ${{\gamma }}_{i}$ confirms that individuals who engage in self-training also show greater retention. The positive correlation between retention rates ${\alpha }_{i}$ and the learning rates ${\beta }_{i}$ is interesting as it suggests that those participants who best respond to training show the most retention. A possible explanation is that training in participants with large ${\beta }_{}$ can bring the participants “above threshold” (see text at the end of results below and in Additional file 1: Fig. S2D), in which case retention is high.

### Comparison with simpler models

The model comparison results show strong evidence that all three main terms in the state-space equation (retention, learning, and self-training) are required for a good fit for both DOSE and EXCITE datasets. Omitting the learning term has the biggest effect in worsening the fit in both datasets, followed by the retention term and the self-training term. Not surprisingly, given the diversity of the MAL trajectories, the population-level model (using only non-individual parameters, i.e., fixed effects) performed the worst based on both the WAIC (588 instead of 297 for the best model) and *elpd_diff*. For details, see Additional file 1: Table S1A for DOSE and Table S1B for EXCITE.

Finally, we compared the model with two static Bayesian regression models: a linear model of time (Eq. 2) and a modified logistic model (see Methods) for both datasets. The logistic model performed relatively well compared to other models, such as the dynamic model without learning, but still largely worse than the full model and the dynamic model without self-training. This illustrates that “fixed” (non-dynamical) models, which cannot simultaneously account for the effects of learning and retention, respectively, cannot account well for our rehabilitation data.

### Evaluation of forecasting accuracy on unseen data: increasing accuracy and precision with additional outcome data

**Fig. 2:**
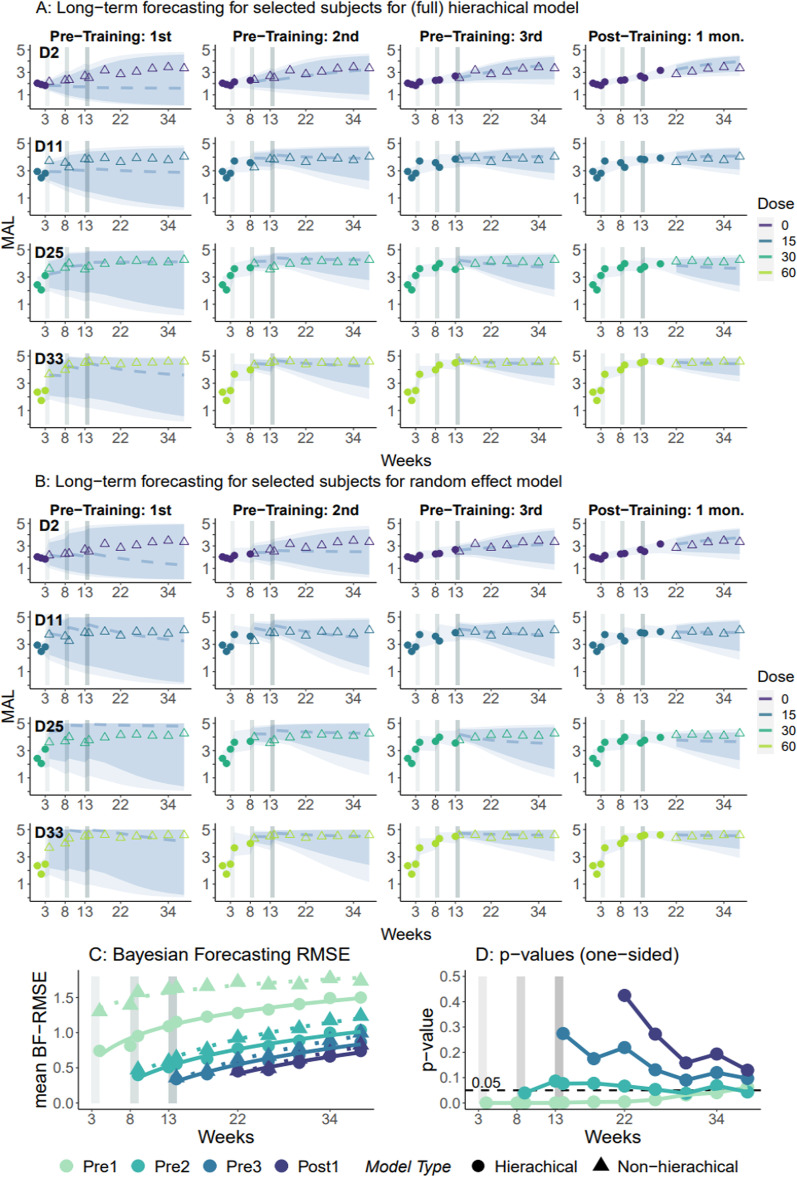
Accuracy of individual long-term forecasting. **A**,** B** Examples of individual predictions for different amounts of incoming data with population priors for four participants, one per dose. D2: 0 h. D11: 15 h. D25: 30 h. D33: 60 h, each in four scenarios in which the availability of outcome measures increases for each participant (circles). Note how when MAL data following the 2nd bout of training is available, predictions at 6 months become remarkably accurate. **B** Same as in **A**, but here, we do not use population hyper-priors but weakly-informative priors for each participant. Solid lines: model prediction (posterior median). Blue shaded zones: 90% and 95% prediction intervals (from darker to lighter). Triangles: MAL data measured but not used to update the model. Note how, when compared with hierarchical model in A, the forecast at 6 month was largely inaccurate and imprecise (i.e., large prediction interval) when only the baseline data were included. **C** Comparison of mean BF-RMSEs for each week in the different forecasting scenarios. Dots and Triangles are the mean BF-RMSEs for the hierarchical and random effect (i.e., non-hierarchical) models, respectively. Solid and dotted lines are the best fit lines (log-linear) of the weekly mean RMSEs for the hierarchical and random effect models, respectively. **D** One-sided p-values for the difference in mean RMSEs between the hierarchical and the random-effect models for each forecasting scenario. Note that whereas the hierarchical model improves short- and long-term forecast until the 2nd bout of training, after the 3rd bout of training, predictions with and without the hierarchy become nearly indistinguishable

We then evaluated the long-term forecasting accuracy for new participants in four different realistic scenarios in which the clinician would assess and re-assess predictions as additional outcome data become available (see Methods). Figure 2A shows the hierarchical model’s forecasting fits (median, 90%, and 95% CIs) on four representative participants for the duration of the DOSE trial. The mean BF-RMSE (averaged over participants) at 6 months post-training was 1.36 when only the baseline data were available. It then decreased to 0.91, 0.79, and 0.69 when the MAL data following the 1st, 2nd, and 3rd bouts of training were available. Thus, when MAL data following the 2nd bout of training is available, predictions at 6 months become remarkably accurate, in line with the Minimal Clinically Important Difference for the MAL of ~ 0.5 [32].

Finally, we find that the random-effect model (in contrast to the hierarchical model) was largely inaccurate when only the baseline data were included (see Fig. 2C and p-values between the two models in Fig. 2D). Whereas the benefit of the hierarchical structure is evident when fewer data are available. Indeed, after the 3rd bout of training, predictions with and without the hierarchy become nearly indistinguishable, as can be observed by comparing the individual plots in Fig. 2A–C. In this case, the $p$-values of the pairwise differences are high, notably for short-range forecasts (Fig. 2D). However, interestingly, for long-range forecasts the p-values decrease again for all scenarios, showing the relative advantage of the hierarchical model (although not reaching the 5% significant level). This occurs because as the proportion of missing data increases in long-range forecasts, information sharing across participants becomes once again more advantageous.

### Qualitative model evaluation

Furthermore, we examined whether the HBDM may serve as a model of motor learning that could reproduce previous findings of the DOSE trial [13]: an immediate dose-response, a decrease in efficiency with both additional dose and weeks of training, a negative dose-dependent effect of training on retention, and the threshold above which self-training becomes sufficient to further improve the MAL without supervised training. For this, we simulated the model of Eqs. 1–3 using all posterior samples of the individual parameters for each of the 40 participants in the DOSE datasets. We processed and plotted the predicted MAL data in a similar fashion to our previous study [13]: (i) To compute the short-term efficacy, we computed the changes in MAL due to the three bouts of training. (ii) To compute the dose efficiency, we divided the changes in MAL due to the three bouts of training by each dose. (iii) To compute the decay following training for each dose, we computed the weekly change in MAL in 2-month intervals following training divided by the number of weeks in each interval. (iv) Finally, to determine the threshold above which self-training can further increase outcomes, we fitted a regression model of the weekly changes in MAL from immediately post-training to 6 months post-training as a function of the mean MAL post-training with dose as a factor.

The model reproduced our five previous results. First, as shown in Additional file 1: Fig. S2A, the model replicated the increase in the efficacy of training with greater doses, with a near-linear dose-response relationship. Second and third, the efficiency of training depends on both the weekly dose and the number of training bouts (Additional file 1: Fig. S2B). Increasing the number of hours of training resulted in a decrease in efficiency in an exponential-like fashion. Similarly, across dosages, the first bout of training increased the MAL, whereas the additional bouts became more and more inefficient (Additional file 1: Fig. S2B). Fourth, retention depends on time post-stroke and the dosage of training. For the smallest dose, the MAL increases following training (positive $\varDelta MAL/week)$, with the initial increase larger than in later months. In contrast, for large dosages, the MAL decreases (negative $\varDelta MAL/week)$, with the initial decay larger than the decay in later months (Additional file 1: Fig. S2C). Whereas such an exponential-like convergence is not surprising given our choice of a first-order state-space model (see examples of fit for 0- and 60-h dose in Fig. 1), these results match those of our previous study in which we approximated the retention data with three linear segments of 2 months each [13]. Fifth, these opposite retention results for small and large doses are due to the positive “self-training” effect counteracting the negative effect of increasing dose (the greater the dosage, the more the forgetting), as shown in Additional file 1: Fig. S2D. When the MAL post-training is above a dose-dependent threshold, the MAL keeps increasing, as we previously found with the piece-wise model [13].

## Discussion

Our study presents a novel model to forecast a continuous motor outcome following rehabilitation in chronic stroke survivors. Although previous static models can predict the response to interventions using baseline data, these models cannot account for the changes in the outcome due to training, self-training, and retention in the long-term. Here, we showed that the accuracy of the prediction is much improved by a dynamic forecasting model that incorporates initial responses to training. Our results using data from both the DOSE and EXCITE clinical trials in chronic stroke showed that the best model is a non-linear state-space model derived from our previous research [12, 13] that contains a retention term that depends on the memory at the previous time-step, a learning term as a function of the dose of supervised training, and the self-training term that uses the predicted MAL fed back to the memory model. Despite its relative simplicity, our model clearly captures the dynamics of the MAL in response to and following training in both datasets. In addition, it accounts for a number of phenomena previously observed in the DOSE study [13].

Comparisons of the hyper-parameters between the two clinical trials show smaller learning rates and self-learning rates and higher retention rate in EXCITE. The smaller learning rates can be explained by our previous study showing that the gains due to therapy [13] decrease with larger doses. Since all participants in EXCITE received 60 h of therapy, compared to an average of 23.5 h of nominal therapy for DOSE, it is expected that the learning rates for DOSE will be greater. Another possibility is that the type of therapy in DOSE (ASAP) is more effective than that of EXCITE (constraint induced movement therapy), but a larger group of 60 h ASAP training would be needed to test this hypothesis. Similarly, greater self-learning hyper-parameter for the DOSE group is consistent with our previous findings that self-learning decreases with the dose of therapy [13]. Finally, in our previous study, we also showed that the greater the gains due to therapy, the greater the forgetting, i.e., smaller retention rates [13]. Thus, a possibility is that constraint-induced movement therapy training in EXCITE is less effective than ASAP in DOSE, leading to less retention. A larger study with diverse range of time since stroke (i.e., participants in the acute, sub-acute, and chronic stages), doses, and types of therapy is needed to test these possibilities.

Although HBDM has not yet been used to inform the practice of neurorehabilitation, hierarchical Bayesian modeling has recently been used to model “spontaneous recovery” in the acute and post-acute phases post-stroke across multiple clinical sites [1]. In our model, the hierarchical structure improved predictions both early in training and for long-term forecasts for a given patient compared to a non-hierarchical model (see Fig. 2A) by “borrowing” information between patients. New incoming data, as well as prior knowledge, can be naturally incorporated in our model, yielding an online supervised learning method that continuously improves the predictions. Importantly, in the hierarchical models, learning occurs simultaneously at the individual and population levels: additional data improves the forecast for the current participant and the population overall. In future work, a higher-level site hierarchy can be added when models fit data from multiple sites, as in [1].

In future applications, HBDM can be used in “precision rehabilitation.” Currently, the dose and schedule of rehabilitation are determined based on clinical setting (e.g., in-patient or out-patient), historical precedent, and results from clinical trials. However, with an accurate forecasting model, one can determine optimal schedules of motor training that maximize expected outcomes. For instance, in previous work in motor adaptation [33], we used optimal control theory to determine the schedule that maximizes the mean long-term performance predicted by a non-Bayesian motor adaptation model. Such predictions of the mean are useful in data-rich applications. However, in personalized medicine, and especially in neurorehabilitation, the data are sparse, and the uncertainty of the predicted outcomes is high. In contrast, the current hierarchical Bayesian forecasting model generates a full distribution of the long-term outcomes based on the parameter uncertainty and measurement noise. This allows us to visualize both accuracy and precision of the model predictions with ease.

In contrast to our theory-driven approach, one can envision a purely data-driven approach to forecasting motor outcomes post-stroke using “black-box” models, e.g., [34]. For instance, deep neural networks trained with a large amount of data could yield good or even better accuracy in long-term predictions. Recurrent neural networks could even generate the dynamics of recovery as in our model. Pre-training a model with large amount of population data and fine-tuning the model with individual data (as in “few shots learning”) [35] could generate predictions for each patient. However, because these models contain very large number of parameters (“weights”), they require a large amount of training data and are difficult (if at all possible) to interpret. For these reasons, such models are often less preferred for high-risk, healthcare applications [36]. In contrast, our approach is sample-efficient and interpretable, which allow us to extract an understanding of the mechanism(s) underlying the individualized predictions by leveraging (reliable) clinical data- see also for a related (non-Bayesian) example [37]. Analysis of the model parameters can help clinicians make informed decisions about therapy [5]. For instance, if a patient has a large learning rate but also a small retention rate, then it is predicted that the gains due to additional therapy will be short-lived.^2^ Alternatively, if the learning rate is near zero, then it can be predicted that even large doses of therapy will not yield large gains. In addition, there are no standard techniques for calculating interval estimates with neural networks for prediction problems. Prediction intervals can be estimated using model ensembles, but these methods lack the rigor of our Bayesian approach.

Notwithstanding, there are two main limitations in our study. The first limitation is the use of the MAL which relies on self-reported ratings of the quality of movement across a range of tasks. Nonetheless, it is striking that our simple model well characterizes the dynamics in the MAL outcome in the two datasets, since the MAL score results from an average of scores for 28 activities of daily living. Relatedly, our “motor memory” variable cannot be taken literally as a neural variable, but rather as an aggregate of the multiple motor memories needed to perform the MAL. We note that, in theory, similar models can be used for other outcome measures that show response to treatment. For instance, we could model the change in the Wolf Motor Function Test [25], as this measure of arm and hand function has been shown to increase in response to treatment in the EXCITE trial. A possible difference, however, is the self-training term, which depends on arm use (and not function). Thus, a model for function would need to be complemented with a model for use, as in our previous work [12]. A second limitation is the limited size and relative homogeneity (because of the restricted entry criteria) of the datasets. As a result, a single covariate was included in the model, the initial MAL. Biomarkers derived from transcranial magnetic resonance and brain imaging would allow us to refine predictions, notably soon after stroke when the predictions are poor, in a more diverse population. In addition, the predictions would further improve with a larger number of measurements from each individual. Furthermore, clinical studies that use connected objects and sensors, e.g., [38], would allow the collection of such datasets that, together with a forecasting model like the proposed HBDM, would form the basis of precision rehabilitation for not only arm and hand function post-stroke but also other functions, such as gait rehabilitation post-stroke, or neurologic conditions such as traumatic brain injury or spinal cord injury.

## Conclusion

Precision rehabilitation can potentially transform the practice of neurorehabilitation: the clinician, patient, and insurance company will be able to choose effective treatments based on individual predictions of long-term recovery. However, because of the large variability of stroke, making accurate long-term predictions for individual patients is hard. In addition, because neurorehabilitation is based on the premise that sensorimotor activity improves motor recovery via brain plasticity, the predictions need to account for increases in performance during movement therapy, but also for possible decreases following therapy. In this paper, we showed that an HBDM that incorporates repeated measurements of performance obtained during movement therapy can forecast motor outcomes at arbitrary future time points based on the effects of clinician-supervised training, self-training, and retention. The Bayesian framework generates full probability distributions of the outcome based on parameter uncertainty and measurement noise, and therefore allows visualization of both the accuracy and precision of the forecasts. Finally, the hierarchical structure improves predictions when little data is available for new patients by “borrowing” knowledge from past patients. As a result, clinical decisions can be made, and refined, early in therapy. For instance, for a new patient, the clinician could simulate the proposed HBDM with different schedules of treatment and plan the treatments according to the forecasted outcomes (notably the schedule and dose). Then, as the real-time data from the current patient become available, the treatment plan could be further refined. However, future models will need to incorporate multiple baseline (or real-time) covariates into the HBDM to obtain predictions that are both more accurate and precise.

## Supplementary Information

**Additional file 1.** Results for model comparison, correlation between estimated parameters, and qualitative model evaluation.

## Data Availability

All code for data analysis and examples of simulated data are available at https://github.com/dongzeye/MAL-Bayesian-SSM. The DOSE data are available upon reasonable request to the last author pending IRB approval. The EXCITE clinical data are third-party data. Researchers must contact the EXCITE PI team for request about data availability.

## Footnotes

1 For instance, a (hypothetical) treatment A, which is predicted to increase the outcome by 50% with a 95% CI of 45–55%, may be preferred to treatment B, which is predicted to increase the outcome by 60% with a 95% CI of 30–90%.

2 In this case however, an alternative possibility could be that the therapy itself is inappropriate; perhaps too much feedback was provided during therapy that created a dependence (i.e., guidance hypothesis) and thus patients do not ‘learn’.
