# Supplementary material for: Long-term forecasting of a motor outcome following rehabilitation in chronic stroke via a hierarchical bayesian dynamic model
Source: J Neuroeng Rehabil. 2023 Jun 29;20:83. doi: 10.1186/s12984-023-01202-y (PMC10311775; doi:10.1186/s12984-023-01202-y)
Supplement: Supplementary file 1 — Additional file 1. Results for model comparison, correlation between estimated parameters, and qualitative model evaluation. [file 12984_2023_1202_MOESM1_ESM.docx]

**Additional File 1. Results**

**Model comparison**

|  | *WAIC* | *elpd_loo* | *elpd_diff* | *se_diff* | *95% CI of elpd_diff* | *Pr(better)* |
| --- | --- | --- | --- | --- | --- | --- |
| *Full (best)* | 296.74 | -149.42 |  |  |  |  |
| *No self-training* | 346.35 | -173.76 | -24.35 | 10.14 | (-44.22, -4.47) | 8.19E-03 |
| *Logistic* | 487.55 | -244.83 | -95.41 | 17.79 | (-130.27, -60.55) | 4.07E-08 |
| *No forgetting* | 496.91 | -249.50 | -100.08 | 17.54 | (-134.46, -65.71) | 5.77E-09 |
| *No learning* | 506.92 | -253.95 | -104.54 | 15.14 | (-134.21, -74.86) | 2.51E-12 |
| *Linear* | 512.34 | -256.55 | -107.13 | 18.60 | (-143.58, -70.68) | 4.20E-09 |
| *Fixed Effect* | 588.35 | -294.26 | -144.85 | 19.77 | (-183.6, -106.09) | 1.19E-13 |

Additional Table S1A: Model comparison for DOSE. *WAIC*: Watanabe-Akaike information criterion. The models are ordered from best to worse, as ranked by the *WAIC*. *Elpd*: expected log point-wise predictive density. *Elpd_loo*: the ELPD approximated using Leave-One-Out Cross-Validation (LOO-CV); *elpd_diff*, a measure of the difference in the elpd_loo between two models. *se_diff* is the (estimated) standard error of *elpd_diff*. The false-positive rate is the probability that the best model is worse than the to-be-compared model.  See Methods for details.

|  | *WAIC* | *elpd_loo* | *elpd_diff* | *se_diff* | *95% CI* | *Pr(better)* |
| --- | --- | --- | --- | --- | --- | --- |
| *Full (best)* | 914.58 | -458.13 |  |  |  |  |
| *No forgetting* | 1079.25 | -539.62 | -81.50 | 19.01 | (-118.76, -44.23) | 9.08E-06 |
| *No self-training* | 1181.18 | -591.06 | -132.93 | 20.81 | (-173.73, -92.13) | 8.49E-11 |
| *Logistic* | 1357.74 | -680.91 | -222.78 | 27.34 | (-276.36, -169.21) | 2.22E-16 |
| *Linear* | 1393.45 | -698.18 | -240.05 | 28.04 | (-295.01, -185.09) | 0.00E+00 |
| *No learning* | 1400.08 | -701.61 | -243.49 | 26.89 | (-296.19, -190.79) | 0.00E+00 |
| *Fixed Effect* | 1501.13 | -750.99 | -292.86 | 27.70 | (-347.16, -238.56) | 0.00E+00 |

Additional Table S1B: Model comparison for EXCITE. See Table 2A for abbreviation.

1. Parameter correlations for the DOSE data.


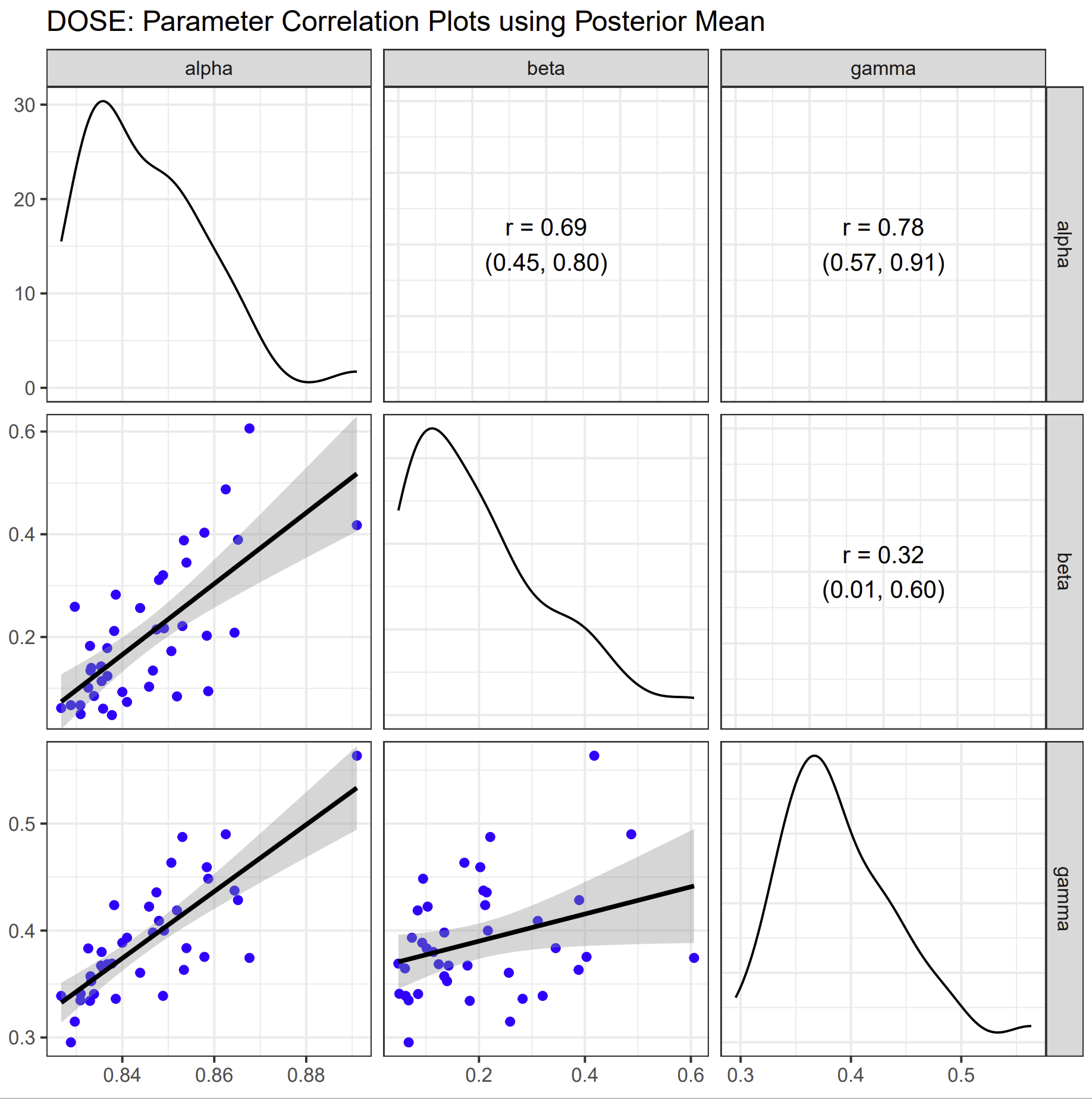


1. Parameter correlations the EXCITE data.


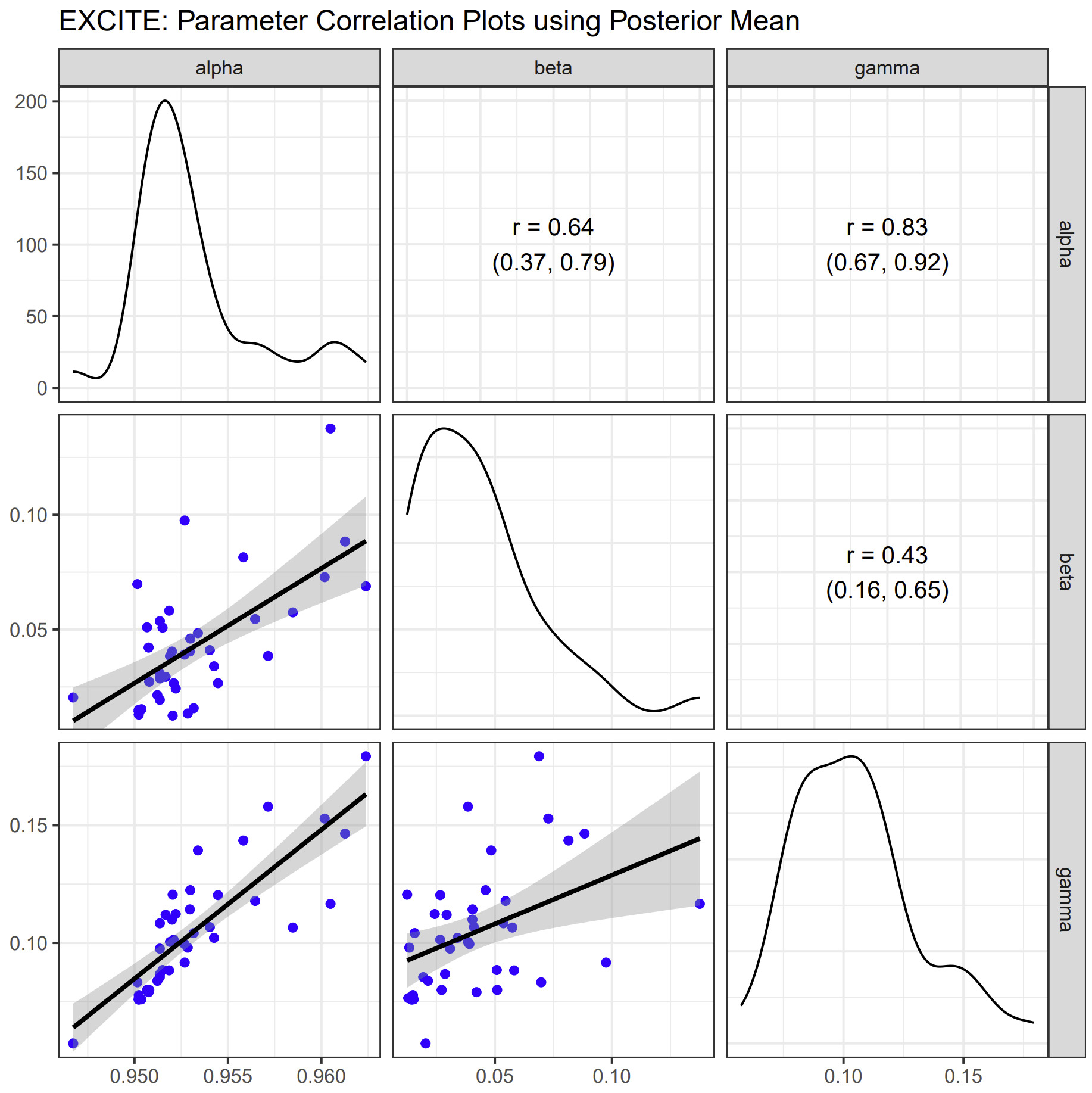


Additional Figure S1: Correlations between estimated retention rates, learning rate, and self-training rates parameters for the individuals in the DOSE trials (A) and the EXCITE trials.

**Qualitative model evaluation**


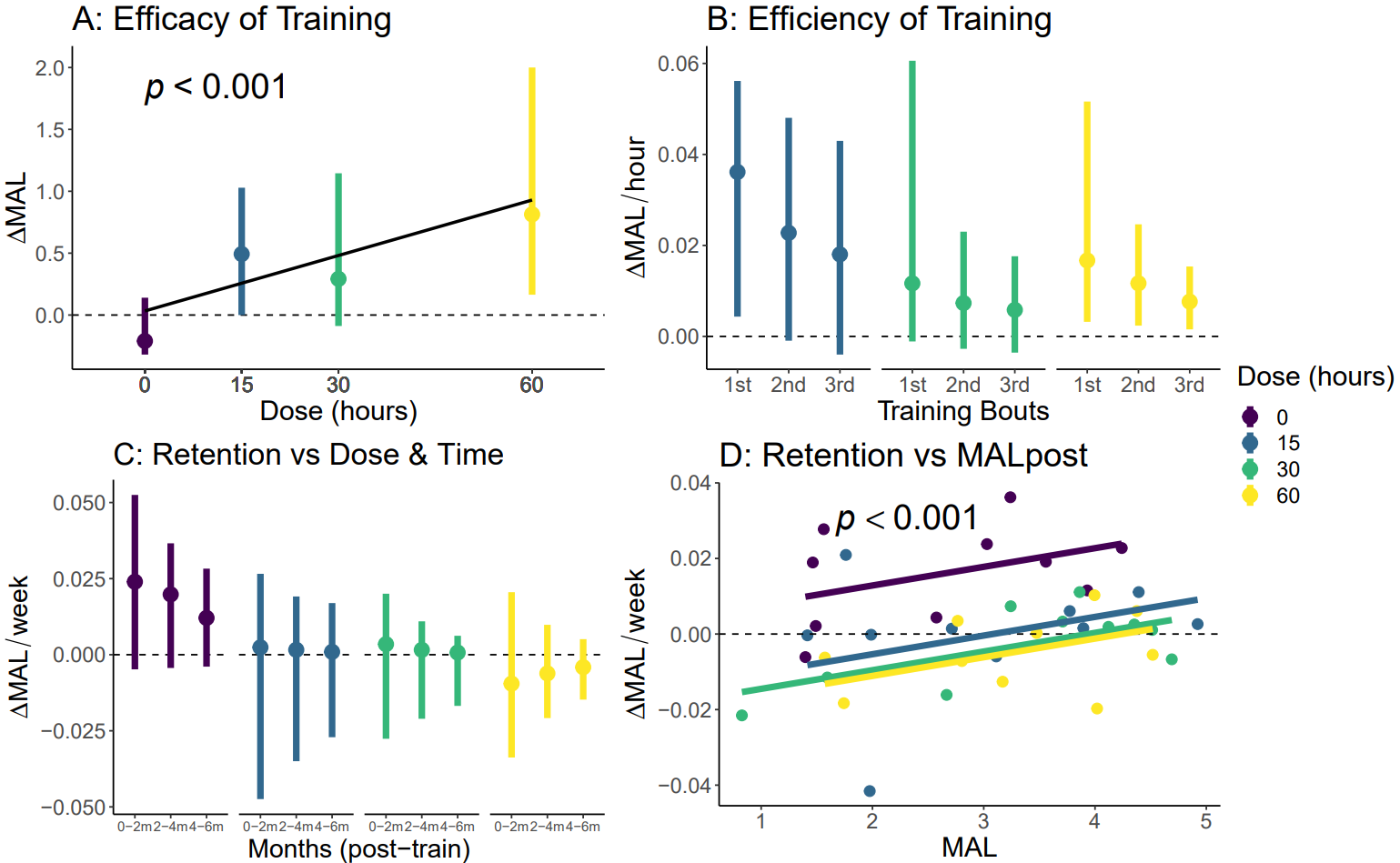


Additional Figure S2: Qualitative validation of the best motor learning model. **A.** Dose efficacy: Effect of dosage on the MAL during supervised training. **B.** Efficiency of training for different training dosages and for the three bouts. Efficiency is computed by the gain in MAL per hour of training. **C.** Effect of time post-training on retention for each dosage: Shown are the forgetting rates in the six months following supervised training as a function of months post-training. **D.** Effect of average post-training MAL on the gain in MAL. The colored lines show retention as a function of average post-training MAL for different dosages, and the colored dots show the individual retention rates. In C and D, the dotted line indicates the retention rate of 0, indicating no change. In A, B, and C, the dots are the population means based on predicted MALs, and the bars the 95% CI.
